# A Qualitative Insight Into Factors Pertaining to Alcohol Consumption Among Young Adult Women During the COVID-19 Outbreak

**DOI:** 10.1177/21676968211067327

**Published:** 2022-04

**Authors:** Amy Montague, Ifigeneia Manitsa, Fiona Barlow-Brown

**Affiliations:** 1 Department of Psychology, 4264 Kingston University London, London, UK

**Keywords:** alcohol use/abuse, young adulthood, women, pandemic, COVID-19

## Abstract

Emerging research suggests young adults, in particular women, may be especially sensitive to changes associated with the COVID-19 outbreak. This study, which is part of an ongoing research project focusing on young adulthood and substance use during the UK COVID-19 lockdown, aimed to provide an in-depth snapshot of factors that young adult women may describe as influential in their alcohol consumption during this period. Virtual semi-structured interviews were carried out on a sample of 12 (23–25 years) women between April and May 2020. The data were analysed through thematic analysis and preliminary findings led to the identification of three themes: (1) Changes to working environment, (2) Limitations on social opportunities and efforts to socialise in a ‘new normal’, and (3) Effects of cohabitation on increased alcohol consumption. The preliminary findings of this study highlight factors relevant to changes in alcohol use during the COVID-19 outbreak in the UK.

## Introduction

Emerging research indicates that more than one in six adults in the U.K. have increased their alcohol use during the COVID-19 lockdown, with approximately 50.4% of those being young adults (Jacob et al., 2021). It has been suggested that this may be because young adults are particularly sensitive to COVID-19-related impacts, since they often occur simultaneously with more normative transitional stressors, such as work, social and educational adjustments (Shanahan et al., 2020). More specifically, COVID-19 disruptions to job and social opportunities may leave this group particularly vulnerable.

Recent research appears consistent with this; for example, Glowcz and Schmits (2020) and Shanahan et al. (2020), both identified young people as being more susceptible and reporting more negative consequences related to lockdown restrictions, such as psychological distress, depression and feelings of uncertainty, than older age groups. Furthermore, Jacob et al. (2021) also attributed their findings to the understanding that COVID-19 social distancing measures, including consequences of lockdown, disproportionately affect young adults. For example, Jacob et al. suggest this group is at increased risk of furlough, leading to a lack of job security and consequent financial anxieties, which may prompt this group to use alcohol to cope. However, Jacob et al., highlight how qualitative work is essential for us to be able to fully understand and appreciate the processes underlying these emerging vulnerabilities. Currently, there is little research which has focused exclusively on young adult samples in relation to their substance use during the COVID-19 outbreak. Both globally and in the UK, these studies have overwhelmingly employed quantitative methods and are also producing conflicting results. Some research with UK samples have reported increases in substance use (Jacob et al., 2021; Niedzwiedz et al., 2021), whereas some report decreases (Evans et al., 2021), with elsewhere following a similar pattern (Glowcz & Schmits, 2020). Furthermore, some report no changes in alcohol consumption from pre-pandemic levels (Charles et al., 2021). Those reporting decreases, such as Glowcz and Schmits (2020), have discussed how this change may reflect restrictions placed on socialising, leading to a lack of drinking opportunities. Glowcz and Schmits (2020) suggested that young adults report decreases in alcohol use for this reason, whereas for middle aged and older adults the opposite is the case, whereby a lack of social connection is leading to increased alcohol use as a form of coping. However, this does not explain why some studies report that young adults are increasing their alcohol use, and it is evident that a more focused investigation is required to explore these discrepancies.

As well as age, there also appears to be gender differences in responses to the pandemic. Women appear to be reporting higher levels of stress and anxiety symptoms and less resilience to stress during this period, than opposite gender counterparts (García-Fernández et al., 2021; Hou et al., 2020; Szabo et al., 2020). Additionally, research which has split by gender has indicated that women may report greater increases in their alcohol use during this period. Jacob et al. (2021) reported that 63.5% of U.K. women indicated having increased their alcohol use during lockdown, compared to 36.5% of men. Alcohol motivation theories (Cooper, 1994) may be relevant in explaining this discrepancy. It has been suggested that coping-motivated drinkers are at a greater risk and most vulnerable for increased alcohol use, as well as solitary drinking during a pandemic (Wardell et al., 2020). Research suggests that women’s drinking is often more coping-related as well as more socially motivated than men’s drinking (Balodis et al., 2009; Norberg et al., 2010); therefore, women may be more vulnerable to elevated alcohol consumption during this period. However, there have been no investigations focusing on women’s alcohol use during this period, using either quantitative or qualitative methods.

Consequently, although this study is part of a larger research project which collected data from a broader sample of both on men and women (all young adults), the present short report focuses only on the data from the women who were interviewed, because of their recognised vulnerabilities during this period. The COVID-19 pandemic is a complex and unique situation which requires a highly granular analysis to unpick the factors which might be driving changes in alcohol use. It was the aim of this research through the employment of qualitative interviewing, to provide a response to the question, ‘How do young adult women describe the COVID-19 outbreak as influential in their alcohol consumption?’

## Method

### Participants

Twelve semi-structured qualitative interviews were carried with young adult women aged 23–25 years (*M* = 24.67, *SD* = 0.65). The ethnicity of the sample largely self-identified as White (*N* = 11), with one participant who identified as Asian. Nine participants were employed; seven had begun working from home and one had their hours reduced because of the COVID-19 outbreak. Three had been furloughed, one of whom was also a student, and one was still part-time employed. Interviews took place virtually via Zoom, between April and May 2020 during the first UK government COVID-19 lockdown period. Participants were recruited via social media advertisement (Twitter, Facebook and Instagram) and identified themselves as drinking on a weekly basis and met the age requirement of 18–25 years. They were also required to have been residing in the United Kingdom since the outbreak began and it was also specified in the eligibility criteria that the individual must not have been diagnosed with a substance use disorder if they wished to apply to participate. Full participant demographics can be seen in Table 1. Pseudonyms were assigned to all the participants. As can be seen in Table 1, participants were asked to describe their average weekly alcohol consumption both at the time of interview and then retrospectively in a ‘normal’ week. Alcohol use was measured in UK units, one UK unit being equivalent to 10 mL (0.34 ounces) or 8 g of pure alcohol. Participants reported either a specified number of units, or number of drinks if they did not have the knowledge to determine their unit consumption. If number of drinks were provided, this was then translated into weekly units to standardise measurements across participants. The research team used standard UK measurements to do this, that is, a single measure of spirit being 1 unit, an approximate measure of beer/cider of unknown strength 2.8 units and likewise, reports of a glass of wine, 2 units.

**Table 1.** Participant Demographics, Including Alcohol Frequencies Before and During the COVID-19 Lockdown.

Participant number	Age (years)	Number of alcohol units per week (approx. UK units)	
		Before the COVID-19 outbreak	During the COVID-19 outbreak
Janice	25	8	6
Sarah	24	13.4	24
Vivienne	25	38.8	38.8
Jo	25	14	40
Jess	25	40.8	6
Anna	23	5.6	0
Khloe	25	7	40
Sophie	24	56	5.6
Marie	25	13.2	70
Elizabeth	25	22.4	66
Jenny	25	27.6	210
Julia	25	56	67.2

## Materials

A semi-structured interview was used in which participants reflected on their own experiences, thoughts and specific issues of interest. The technique allowed researchers the flexibility to refine the interview schedule according to participants’ stories and to explore motives which had not been investigated before (Horton et al., 2004). The questions in the semi-structured interview were based on an interview guide that was developed to examine several factors which could influence alcohol use during the COVID-19 outbreak. Extracts of the interview schedule are available in the online supplementary material. Each interview lasted approximately 30–45 minutes. This study received ethical approval from (included in the title page).

### Data Analysis

All interviews were recorded and then transcribed. The process of transcription was shared between authors and an independent, university secured, transcription service. Once transcribed, data were analysed through the steps of Thematic Analysis (TA) (Braun & Clarke, 2006). Thematic analysis focuses on the identification of patterns of meaning across a dataset. More precisely, it consists of the following six phases: (1) familiarization with the data, which refers to the reading of the data and the identification of repeated patterns in the dataset, (2) initial coding, which focuses on the extraction of the most fundamental information and the emergence of important features from the dataset, (3) searching for themes, which involves the formation of themes and sub-themes based on the initial codes, (4) reviewing themes, which refers to the re-evaluation of the themes against the dataset and the confirmation that they create a coherent story, (5) defining and naming themes, which includes the detailed analysis of each particular theme and its sub-themes and the allocation of a name to each theme/sub-theme and (6) producing the report, which involves the write-up of the data (Braun & Clarke, 2006).

The first two authors coded the data independently of each other. Separately, they followed the first two steps of TA. Firstly, both authors familiarised themselves with the data through multiple readings of the transcriptions. After this, line by line coding took place for each participant, using MS Word and Excel software. During the coding process, through the participants’ descriptions of their alcohol use, authors searched for parallels across the dataset. Once coding was complete, author three subsequently examined the codes to confirm they were coherent and unbiased. Finally, as a collective team, authors one, two and three completed steps three, four and five whereby examination of the earlier identified codes took place to consolidate themes. Themes were continually reviewed throughout this process to ensure their relevance to the research question. Additionally, points of difference were discussed throughout the process of developing themes, until a consensus was reached by the whole team.

### Findings

Through thematic analysis, three themes were identified: (1) Changes to working environment, (2) Limitations on social opportunities and efforts to socialise in a ‘new normal’, and (3) Effects of cohabitation on increased alcohol consumption. All participants described changes to their daily schedule, stemming from the COVID-19 outbreak, as pertinent in changes in their alcohol consumption. These changes were mainly associated with the lack of a ‘normal’ working environment, sudden limitations on social opportunities external to the home environment, attempts to socialise during a ‘new normal’, and finally, the impact of cohabitation with family members, partners and friends during lockdown. In parentheses after participant quotes are the participant’s pseudonym, age and average weekly alcohol consumption (UK units of alcohol) prior to the outbreak, to during lockdown at the time of interview.

### Changes to Working Environment

Eleven of the 12 participants reported substantial changes to their usual employment because of the COVID-19 outbreak. Therefore, majority of the sample had experienced some form of disruption to their daily structure and routine, as well as changes to their job security. Most often, these employment-related changes and the process of adjusting to a new working routine were critical in explaining the changes in their alcohol use. Of these 11 participants, four participants reported a decrease in their alcohol use, whereas seven an increase. Predominantly, for those who reported increases in alcohol use these changes were implicated in several ways. For example, some participants, such as Khloe, discussed using alcohol to cope with financial anxieties instigated by a reduction in her working hours:

“I think I am a little bit more stressed… pay is a little bit less now that I am working less ... I am stressing out a little bit and as I am drinking more….” (Khloe, 25 years, 7 units a week pre-lockdown to 40 units a week during lockdown).

It was evident that Khloe was feeling distressed during lockdown because of the financial and employment insecurities prompted by the COVID-19 outbreak. However, changes in workload were also implicated in increases in drinking for other reasons. Primarily, participants discussed how a decrease in their volume of work, as well as lowered expectations from their workplace and changes in working hours due to working from home, meant they had more leisure time throughout the week. They described having fewer restrictions on when they could drink, and having no, or reduced, working hours meant there was less to prevent them from drinking; also, the distinction between weekday and weekend was becoming less pronounced “*I think every evening kind of feels... like a weekend evening because you know you have nothing to get up for in the morning… I think you feel more relaxed to have more than a glass of wine because you are not worried about, I need to have a clear head for the morning.” (*Julia, 25 years, 56 alcohol units a week pre-lockdown to 67.2 units a week during lockdown*)*

As well as a decrease in participant’s current workloads, participants who were furloughed also described having more leisure time, which in turn had a direct impact on their alcohol consumption. The following furloughed participant, Marie, was one who reported a significant increase in their number of weekly units. Similarly, they attributed this increase to having less concern about a hangover the following day because they were not required to be at work:

“I guess also because I haven’t got work and I haven’t got set shifts for the next day, then I don’t need to worry so much about how much I am drinking or how much I am sleeping.” (Marie, 25 years, 13.2 units a week pre-lockdown to 70 units a week during lockdown).

The importance of a routine was something many participants discussed; the significance of this is articulated by Jenny in the following quote. Jenny substantially increased her alcohol intake during this period, and stressed the importance of a working routine to give her more control over her alcohol use:

“I think the control of the drinking is more difficult… I think for most people, even family, it’s just the reasons you don’t tend to drink is because you have got your routine. Having that taken away… that is a big thing.” (Jenny, 25 years, 27.6 units pre-lockdown to 210 units during lockdown)

As highlighted by Jenny, and emphasised throughout this theme, many of the participants who discussed an increase in their alcohol use would attribute this to changes to the structure of their day, usually associated with employment changes. These changes were often related to the use of alcohol to cope with financial anxieties and employment instability. Also, since most participants had been working from home, and had reduced or more flexible hours, they had more leisure time and fewer restrictions on their alcohol use.

### Limitations on Social Opportunities and Efforts to Socialise in a New Normal

As well as changes to daily structure, it was evident throughout participant discussions that many of the women closely associated alcohol with social events, with nine of the women reporting their alcohol use as being primarily socially motivated. Due to the COVID-19 outbreak, there was a lack of opportunity to participate in social events, owing to lockdown restrictions. This was directly related to a reduction in four of the participants’ alcohol use, for example, Anna:

“For instance, sat at home with just myself and (housemate), I wouldn’t drink. However, if I was sat at home myself, (housemate) and all our friends, I would drink…it’s like if the environment is right, it has to be a mix of do I want to drink in this environment yes or no.” (Anna, 23 years, 5.6 units pre-lockdown to 0 units during lockdown)

Through discussions with Anna, a participant who consumed a small amount of alcohol weekly pre-pandemic, the lockdown restrictions had led her to stop drinking alcohol completely. Anna described how the lockdown had meant there was no longer a social outlet for their alcohol use, and they were unable to experience a communal atmosphere when drinking. They describe how drinking became less appealing and they felt demotivated to consume any alcohol during the lockdown period. Janice had a similar perspective and described how this was impacting her wellbeing:

“At the weekends I would love to go socialising and for a drink with friends and that’s not happening, I’m realising how much purpose I held in meeting other people, and socialising… how it made me feel good.” (Janice, 25 years, 8 units pre-lockdown to 6 units during lockdown)

Janice, who also reported a decrease in her alcohol use throughout lockdown, described how no longer being able to drink alcohol with her peers at weekends left her feeling dejected. She highlighted how drinking while socialising was an important aspect of her weekly routine, and lockdown had stopped that from occurring. Although she had decreased her alcohol use, being unable to socialise, which would occur alongside drinking, was described as negatively impacting her wellbeing.

There were also distinct dialogues across these participants about the role that ‘virtual socialising’ played in their alcohol consumption. Some participants discussed how, although they consumed very little alcohol throughout lockdown and had significantly decreased their weekly drinking, if they did drink it was solely during virtual social events *“Yes. We did a quiz last night and that was probably the most I’ve drank since lockdown...”* (Jess, 25 years, 40.8 units pre-lockdown to 6 units during lockdown). Although socialising alongside drinking could no longer occur in an external location, attempts were still made by participants to align drinking with their social motives, using other methods such as virtual pub quizzes. As can be seen in the following quote from Sophie, participants often viewed drinking at virtual social events as a method of continuing their pre-pandemic behaviours. Sophie highlighted how she expected that she would continue her drinking behaviours throughout the lockdown. However, these new ways of social drinking did not always align with her expectations:

“At the beginning, I was like oh well, I drink a lot and so that is sort of what I do. You are sort of trying to still make it fun and be upbeat. Whereas now, I get less from the upbeat.” (Sophie, 24 years, 56 units pre-lockdown to 5.6 units during lockdown)

Therefore, as highlighted in this theme, participants whose alcohol use motivations aligned closely with social-specific goals decreased their alcohol use. These participants attributed this change to the inability to socialise alongside their alcohol use, because of the pandemic restrictions. Although participants made attempts to socialise alongside their alcohol use in a similar way to their pre-pandemic behaviours, such as by participating in virtual events, for most this did not provide the necessary outlet and was not providing participants with the desired social rewards. Furthermore, the limiting of social activities was having impacts on reported wellbeing through the lockdown period.

### Effects of Cohabitation on Increased Alcohol Consumption

Of the nine women who reported social motivations for alcohol use, five of them reported an increase in alcohol use and identified people who they lived with during lockdown as contributing to this. For example, some of these participants related their family’s lockdown drinking very closely to their own, “*Then I came home, and it was like this is weird. Let’s drink... I definitely had a very heavy first night… Definitely have made do by doing lots of deliveries for alcohol... We have definitely been drinking.”* (Marie, 25 years, 13.2 units pre-lockdown to 70 during lockdown). When describing the uniqueness of the pandemic, Marie explained how drinking was a way for herself and her family to cope with the unfamiliarity of the pandemic. Similarly, as in the previous theme, the continuation of alcohol use as a pre-pandemic behaviour was a useful way for Marie and her family to preserve some normality during this unusual period. Some participants, such as Elizabeth, who saw an increase in their alcohol use during the lockdown, described how drinking was an enjoyable family activity which could take place in the home, at the end of a working day:

“A lot of that time I wake up and me and my brother, my brother’s wife, is like let’s not drink today. When it gets to five o’clock it’s like, what do we do now? That happened yesterday and we were just like let’s have a gin and tonic [Laughs]." (Elizabeth, 25 years, 22.4 units pre-lockdown to 66 units during lockdown)

However, family drinking was not always discussed positively. One participant described how increased alcohol consumption was leading to a great deal of volatility in their household. Jenny, who reported a significant increase in units consumed per week mentioned that her family, who had similarly increased their drinking during the lockdown period, stated that due to their increased drinking there was conflict between her and her family members:

“If I had a drink for example on a Thursday or a Friday, I may be the only one... Now, my dad will have already had a drink and then I am having a drink and then my mum has had a drink... It’s just the number of us that are doing it… it has become noticeably more obvious that we will argue with each other.” (Jenny, 25 years, 27.6 units pre-lockdown to 210 units during lockdown)

Jenny described how as all of the adults in her household had increased their drinking, and she felt that everyone’s moods had been impacted. She mentioned alcohol increasing hostility, which was leading to many family arguments, which were particularly stressful and challenging for her to manage during an already challenging period.

Some participants also described how cohabitating during lockdown with significant others, as well as friends, impacted on their alcohol use. For instance, the direct impact of flatmates’ alcohol norms on participants’ drinking behaviours was mentioned, *“…with my flatmates probably, if any of us starts drinking the rest of them follow suit...”.* (Jo, 25 years, 14 units pre-lockdown to 40 units during lockdown). Likewise, participants’ partners’ drinking norms also had an influence. Sarah, who increased her alcohol use during lockdown, explained that both her and her partner were now working from home and although she might not of had a desire to drink, her partner’s drinking could act as a prompt for her to have a drink:

“…yes, it’s definitely something we would do together… I’d be very easily influenced by my partner suggesting to me to have a glass of wine…I would be more likely to say yes, even if I wasn’t much feeling the urge.” (Sarah, 24 years, 13.4 units pre-lockdown to 24 units during lockdown)

It was evident from Sarah’s description that her partner played a key role in encouraging her alcohol use during lockdown. With both herself and her partner working from home, she felt herself more tempted to have a drink than if she had been working alone. Like Sarah, several participants felt that the individuals who they were sharing a home with during lockdown were influential in their increased alcohol use.

## Discussion

The broad aim of this research was to investigate how the experiences of national lockdown and the COVID-19 outbreak impacted on alcohol use in a young adult UK sample. Preliminary results allowed for analysis of a women-only sample. A preliminary investigation of a women-only sample is worthwhile because of their likely vulnerability during the lockdown period as a consequence of their drinking motivations (García-Fernández et al., 2021; Hou et al., 2020; Jacob et al., 2021; Szabo et al., 2020).

As noted, current research investigating substance use in the UK, as well as more globally, are reporting conflicting evidence and also are solely employing quantitative methods (Charles et al., 2021; Evans et al., 2021; Glowcz & Schmits, 2020; Jacob et al., 2021; Niedzwiedz et al., 2021; Winstock et al., 2020). Although these quantitative findings are useful, they do not allow for an understanding of the experiences of individuals during such a period. This study provides a response to this by offering an insight into individual experience during a pandemic and how this subsequently affects alcohol use. The main themes which were identified through thematic analysis were (1) Changes to working environment, (2) Limitations on social opportunities and efforts to socialise in a ‘new normal,’ and (3) Effects of cohabitation on increased alcohol consumption. Our findings are consistent with the conflicting outcomes of previous research, in that some people are increasing their alcohol use, whereas some are decreasing it (Charles et al., 2021; Glowcz & Schmits, 2020; Niedzwiedz et al., 2021). However, by using qualitative methods this research suggests factors which could be driving these differences.

The findings highlight the importance of acknowledging individual factors for understanding why an individual reports either increasing or decreasing their alcohol use during a pandemic. Through participant conversations, it became apparent that alcohol motives played a key role here. More specifically, those who were more coping-motivated were more likely to increase their alcohol use to manage pandemic-related stressors, which were largely employment-focused. This supports the suggestion made by Jacob et al. (2021) who attribute the increase in young people’s drinking as a form of coping with their increased risk of being furloughed and subsequent financial and employment instability. Additionally, non-pandemic alcohol motivation research suggests that those who are coping motivated, are more likely to develop alcohol-related problems (Merrill et al., 2014). This therefore raises concerns that we may be likely to witness an increase in alcohol-related problems, which have been prompted or exacerbated by the pandemic for these individuals.

However, in this research, it was not only coping with financial and employment instability which led to increases in alcohol use, but also changes in daily structure and routine caused by changes to their employment. Through emerging research summarised in Gonçalves et al. (2020), lockdown is requiring the employment of much higher levels of self-monitoring, organisation and self-discipline, which inevitably some will find challenging. Without sufficient employment of these, it is believed individuals would be more susceptible to increased alcohol use. Gonçalves et al. (2020) further emphasised the importance of routine and structure, by acknowledging the common practice of targeting these factors during substance use disorder treatment to help reduce alcohol use. Furthermore, for those who were more socially motivated the environment which they found themselves in during lockdown was pertinent in determining whether their drinking would increase or decrease. Those who reported socialising alongside drinking with those they were living with during the lockdown reported increases in use. This highlights the relevance of socialising and social influence in prompting drinking during lockdown, and the impact of who the individual spent their time at home with. Likewise, there were also those who felt they had no social outlet of any kind for their drinking and subsequently reported decreases in use. This is consistent with Glowcz and Schmits (2020) who stressed the importance of social restrictions in reducing alcohol consumption by young adults in such a period. As demonstrated, for those who are more socially motivated, the importance of an individual’s household environment, during the context of a lockdown is key to explaining possible changes in their alcohol use. Prior research has suggested social motivations are associated with greater alcohol consumption (Van Demme et al., 2013), therefore, it is not surprising that those who socialised within their home environments, reported increases in use and those who did not, decreases. These findings do pose questions relating to the response of those who are socially motivated in the face of easing of restrictions and adjustment back to a ‘normal’ life. With there being a clear relationship between social-motivation and greater consumption of alcohol use, it may be that we will see an acute increase in alcohol consumption in this adjustment period as individuals are able to socialise once again. Interestingly, the changes in alcohol use discussed in this theme were prompted by consequences of the lockdown period, as opposed to concerns and distress relating to COVID-19 itself. For example, as opposed to worries of family members or themselves becoming sick, participants more readily discussed their job and routine changes. This finding suggests contextual elements of the pandemic were much more prominent in participants alcohol use, then distress directly related to the global crisis. This could be explained by the age of the sample, with research suggesting older age groups report higher risk perceptions of COVID-19 infection (Niño et al., 2021), as well as the motivational alcohol profiles of the participants, with those being more socially motivated being less likely to use alcohol to cope with distress.

These findings are especially pertinent in the context of a woman only sample, supporting emerging research which suggests that women may be particularly vulnerable during this period (García-Fernández et al., 2021; Hou et al., 2020; Szabo et al., 2020; ). This also supports findings that women may be more coping motivated in negative situations and with aversive emotions than men (Norberg et al., 2010), especially as results suggested that 60% of participants reported an increase in their alcohol use associated with pandemic-related changes, such as employment uncertainty and adjustments to new employment norms. Moreover, as predicted given the focus on women, social changes were particularly pertinent in the discussions. This supports research which has highlighted the importance of social motivations for alcohol use among this group (Balodis et al., 2009). With many participants describing social incentives for their alcohol use, specific patterns of behaviours were identified for how their alcohol use had adapted to the context of a lockdown.

These preliminary findings provide a unique insight into the impact of lockdown and the outbreak of COVID-19 on alcohol use for a sample of young women. However, it is important to consider limitations of this research. One limitation is the lack of diversity within the sample. The participants were almost exclusively White British and from the London area, limiting generalisability. Likewise, this study aimed to provide an overview of experiences of young adults, however, participant ages ranged from 23 to 25 years, which does not allow for investigation of those at the beginning of young adulthood. Furthermore, this research only focused on self-identified ‘low-risk’ drinkers. With those who are coping-motivated likely to be more vulnerable during a pandemic, it is also important for future research to explore more at-risk alcohol users in such periods. Additionally, participant eligibility encompassed anyone who consumed alcohol weekly, leading to considerable variation in the frequency of alcohol use across the sample. Finally, the setting and timing of this study are likely to have been influential. As the pandemic is ever changing, these results provide a unique snapshot at the onset of the outbreak in the U.K., and it is important to consider the specific social and political climate of this period when assessing these findings.

This research highlights unique factors which have prompted increases or decreases in alcohol use during the COVID-19 lockdown. From these findings, the authors encourage the use of alcohol motivation-theories to understand potential changes in alcohol use during the context of a pandemic. By focusing on alcohol use motivations, vulnerabilities for increased in alcohol use during a pandemic can be more easily acknowledged for women and young adults alike. These factors may be particularly useful for future public health interventions to promote safe alcohol use during national disasters. The findings also provide direction for quantitative work which focuses on alcohol use during and adjusting to life after a pandemic. When working with young adult samples, alongside the inclusion of motivational measures, the authors recommend including survey measures to capture pandemic-related employment and job stress, as well as environmental factors such as participants home environment during lockdown periods. Beyond the specific effects of the pandemic and lockdown on alcohol use, the current study has implications for other circumstances. The experience of disjointed routine led to changes in alcohol use, and it seems likely that such an outcome could occur in other situations where the working week loses structure, such as through unemployment or working from home, which is also relevant outside of the pandemic context. COVID-19 presented a unique opportunity to witness the effects of an extreme situation, but the conclusions we can draw spread beyond this unique situation.

## Supplemental Material

sj-pdf-1-eax-10.1177_21676968211067327 – Supplemental Material for A Qualitative Insight Into Factors Pertaining to Alcohol Consumption Among Young Adult Women During the COVID-19 Outbreak Click here for additional data file. Supplemental Material, sj-pdf-1-eax-10.1177_21676968211067327 for A Qualitative Insight Into Factors Pertaining to Alcohol Consumption Among Young Adult Women During the COVID-19 Outbreak by Amy Montague, Ifigeneia Manitsa, and Fiona Barlow-Brown in Emerging Adulthood
