# Supplementary material for: A Qualitative Insight Into Factors Pertaining to Alcohol Consumption Among Young Adult Women During the COVID-19 Outbreak
Source: Emerg Adulthood. 2022 Apr;10(2):511–8. doi: 10.1177/21676968211067327 (PMC8808132; doi:10.1177/21676968211067327)
Supplement: sj-pdf-1-eax-10.1177_21676968211067327 – Supplemental Material for A Qualitative Insight Into Factors Pertaining to Alcohol Consumption Among Young Adult Women During the COVID-19 Outbreak [file sj-pdf-1-eax-10.1177_21676968211067327.pdf]

Supplementary Material

Interview schedule

1. On a weekly basis, do you smoke, drink alcohol, or both?
2. On an average day, can you think of any factors which might lead you to have a cigarette, or an alcoholic drink?
3. Thinking about the COVID-19 outbreak, how did you react to the rapid spread of the virus worldwide? 3.1. *What were your concerns, and did they change from when the spread started to now?*
4. Would you describe the COVID-19 outbreak as changing your life? If yes, can you give me some examples in ways which your life has changed?

Self-isolation

1. Have you, or anyone you live with been sick during the COVID-19 outbreak? And did this lead to a period of self-isolation? *(clarify - self-isolating being the process of placing oneself in isolation, not leaving home for any reason).*

Government lockdown

2. Did your daily routine change upon the government lockdown restrictions? Can you give me some examples of changes that stand-out for you?

Family

3. Since the restrictions were put in place, how would you describe your relationship with your family?

Friends

4. What about relationships with your friends?

Media

5. What do you think of the media's response to COVID-19?

Work

6. Were your studies/work effected by the outbreak? If so, how?

Summary Questions

7. Since the COVID-19 outbreak, would you say the number of drinks (and/or) cigarettes which you mentioned earlier, has changed? What have they change too?
